# Weighted Blanket Load and Sleep Outcomes During Daytime Napping and Overnight Sleep: Two Randomized Crossover Studies

**DOI:** 10.3390/healthcare14203416

**Published:** 2026-10-13

**Authors:** Ilias Ntoumas, Nikolas Antoniou, Nikos Bourganos, Stergioula Stylou, Afroditi Kotsi, Konstantinos Zarogiannis, Giorgos Katsiaribas, Christina Simou, Paraskevi Gkiata, Cody Garett Durrer, Andreas Flouris, Christoforos D. Giannaki, Christina Karatzaferi, Giorgos K. Sakkas

**Affiliations:** 1 Department of Physical Education and Sport Science, School of Physical Education, Sports and Dietetics, University of Thessaly, 42100 Trikala, Greece; intoumas@uth.gr (I.N.); adoniounikolas@gmail.com (N.A.); nickbourga@gmail.com (N.B.); stellastyloy@gmail.com (S.S.); afroditikwtsi@gmail.com (A.K.); konzarogia@gmail.com (K.Z.); gkatsiaribas@gmail.com (G.K.); xristinasimou6@gmail.com (C.S.); gkiata.vivi@gmail.com (P.G.); aflouris@uth.gr (A.F.); ck@uth.gr (C.K.); 2 Centre for Physical Activity Research, Copenhagen University Hospital–Rigshospitalet, 2100 Copenhagen, Denmark; cody.garett.durrer@regionh.dk; 3 Department of Life Sciences, School of Life and Health Sciences, University of Nicosia, Nicosia 2417, Cyprus; giannaki.c@unic.ac.cy; 4 Research Centre for Exercise and Nutrition (RECEN), University of Nicosia, Nicosia 2417, Cyprus

**Keywords:** weighted blanket, daytime napping, polysomnography, sleep latency, sustained sleep efficiency, deep pressure stimulation, relaxation

## Abstract

**Background/Objectives**: Weighted blankets provide deep pressure stimulation and may support relaxation, sleep initiation, and sleep continuity. However, the optimal acute blanket load across different sleep contexts remains unclear. This study examined the acute effects of 7 kg, 9 kg, and 14 kg weighted blankets on daytime napping and overnight home sleep in healthy adults. **Methods:** Two complementary randomized crossover protocols were integrated into one manuscript. In Protocol A, 20 healthy young adults completed four randomized 30-min laboratory nap conditions: control/sham blanket, 7 kg, 9 kg, and 14 kg weighted blankets. In Protocol B, 20 healthy young adults completed four randomized overnight home sleep conditions: personal blanket control, 7 kg, 9 kg, and 14 kg weighted blankets. All conditions were separated by one-week washout intervals. Portable EEG/EOG polysomnography was used to assess nap and sleep architecture. Relaxation state, handgrip strength, blood pressure, heart rate, and skin/ambient temperature were also assessed where applicable. **Results**: During daytime napping, no statistically significant differences were observed across conditions for sleep outcomes. During overnight home sleep, the original gender-adjusted repeated-measures model indicated a significant condition effect on sustained sleep efficiency [F(1.929, 9.646) = 14.223, *p* = 0.001, η^2^p = 0.740], with higher values under the 9 kg and 14 kg conditions compared with control. However, a sensitivity analysis excluding gender, using the same seven complete cases, did not confirm a significant overall condition effect [F(3, 18) = 0.630, *p* = 0.605]. Systolic blood pressure decreased after overnight sleep with the 9 kg blanket, while skin and ambient temperatures remained unchanged. **Conclusions**: Weighted blankets did not significantly affect objective sleep outcomes during the daytime nap protocol. During overnight sleep, the original gender-adjusted model suggested a potential effect on sustained sleep efficiency; however, this finding was based on only seven participants with complete data and was not reproduced in either the sensitivity analysis excluding gender or the non-parametric Friedman analysis. The remaining objective sleep outcomes did not differ significantly across conditions. Accordingly, the sustained sleep efficiency finding should be considered exploratory and model-dependent rather than confirmatory evidence of an effect of weighted blanket load on overnight sleep. Larger and longer-term studies are needed before conclusions regarding the overall effectiveness of weighted blankets for sleep can be drawn.

## 1. Introduction

Sleep is essential for physical restoration, cognitive performance, emotional regulation, and cardiometabolic health. Insufficient or disturbed sleep is common globally and is associated with adverse health outcomes and economic burden [1,2,3,4]. Stress and heightened physiological arousal can impair both sleep initiation and sleep maintenance, making non-pharmacological strategies that promote relaxation clinically and practically relevant [5,6].

Daytime napping and overnight sleep represent different but complementary opportunities to improve recovery. Short daytime naps can improve alertness, cognitive performance, fatigue, and readiness for subsequent physical or mental tasks [7,8]. Overnight sleep, in contrast, reflects the main restorative sleep period and is strongly influenced by sleep continuity and fragmentation. Therefore, the ability to use the same intervention across different sleep contexts, including both daytime napping and overnight sleep, may enhance its practical applicability.

Weighted blankets are designed to deliver deep pressure stimulation, a gentle and distributed tactile input that may promote parasympathetic activation and reduce arousal [9,10]. Previous studies suggest that weighted blankets may reduce anxiety, improve subjective sleep quality, and increase pre-sleep salivary melatonin in young healthy adults or clinical groups [11,12,13,14]. However, evidence remains limited regarding the optimal blanket weight, particularly under objective polysomnography and across different real-world sleep contexts.

The present integrated manuscript combines two complementary randomized crossover protocols conducted by the same research group. The first examined the effects of 7 kg, 9 kg, and 14 kg weighted blankets on a 30-min daytime nap under controlled laboratory conditions. The second examined the same blanket loads during overnight home sleep using objective polysomnography and temperature monitoring.

The aim was to provide a unified interpretation of whether weighted blanket load acutely affects sleep initiation, sleep continuity, relaxation, physiological arousal, and thermal responses.

We hypothesized that weighted blankets would improve sleep initiation and continuity and promote relaxation compared with the control conditions, with moderate blanket loads expected to produce the most favorable responses across both sleep contexts.

## 2. Materials and Methods

### 2.1. Study Design and Participants

This paper is an integrated report of two complementary acute randomized crossover protocols. The daytime nap protocol was registered as “Daytime Napping and Weighted Blankets (Heavy Nap)” under ClinicalTrials.gov identifier NCT06789237 and was approved by the Human Research and Ethics Committee of the University of Thessaly, Trikala, Thessaly, Greece (2329/07/02/2024). The overnight home sleep protocol was registered as “Impact of Weighted Blankets on Sleep Quality (CosyNight)” under ClinicalTrials.gov identifier NCT06842797 and was approved by the Human Research and Ethics Committee of the University of Thessaly, Trikala, Thessaly, Greece (3-1/5.2.2025). All participants provided written informed consent before participation. Protocol A (daytime nap) was conducted in 2024, whereas Protocol B (overnight home sleep) was conducted in 2025.

Because the two protocols had different study settings, participant characteristics, and trial registration numbers, the data were not pooled into a single analysis. Instead, the protocols are presented as two coordinated components of one integrated study: Protocol A, a daytime laboratory nap experiment, and Protocol B, an overnight home sleep experiment.

In Protocol A, 20 healthy adults (22.05 ± 1.98 years; 13 women and 7 men) were recruited from the student population of the University of Thessaly. In Protocol B, 20 healthy adults (22.0 ± 1.2 years; 10 women and 10 men) were recruited as volunteers from the local community of Trikala, Thessaly, Greece. Inclusion criteria for both protocols were age > 18 years and capacity to participate in the sleep procedures. Exclusion criteria included history of insomnia or other known sleep disorders, mental illness, history of epilepsy, and any acute or chronic condition that would limit participation; the overnight protocol additionally excluded opioid medication use.

### 2.2. Interventions and Procedures

**Protocol A** was conducted at the Lifestyle Medicine Laboratory, Department of Physical Education and Sport Science, University of Thessaly, in Trikala, Thessaly, Greece. Prior to the initiation of the study, participants completed a series of questionnaires and assessments related to their overall health status. For neurophysiological monitoring, participants were connected to a portable EEG/EOG system (HST-mit-tablet, SOMNOmedics AG, Randersacker, Germany) to assess sleep architecture and physiological responses throughout the intervention.

Before and immediately after each experimental condition, participants completed the Relaxation Sensation Questionnaire (RSQ) to evaluate subjective relaxation levels. In addition, maximal voluntary muscle strength was assessed using three maximal handgrip attempts with the dominant hand using a digital handgrip dynamometer (MAP 80 K1, KERN & SOHN GmbH, Balingen, Germany). Vital signs, including heart rate and blood pressure, were measured using an automated blood pressure monitor. All assessments were performed while participants remained in the supine position.

Participants attended four experimental sessions separated by one-week intervals. The one-week interval was used to separate successive acute exposures to different blanket loads and to reduce the potential for carryover effects between experimental conditions. During each session, participants rested in the supine position for 30 min under one of four randomized conditions: a cotton control/sham blanket without additional weight, a 7 kg weighted blanket, a 9 kg weighted blanket, or a 14 kg weighted blanket. The three blanket weights (7, 9, and 14 kg) were selected based on commercial availability. These distinct, fixed loads were used to explore potential weight-dependent differences in the measured outcomes rather than to test established weight recommendations or individualized prescriptions based on participants’ body mass. For each participant, another member of the research team used a random draw to assign the four blanket conditions to numbers 1–4. At the first visit, the participant stated their preferred order of the four numbers, and the blanket conditions were then administered according to the corresponding number-to-condition assignment. A new assignment was drawn for each participant. All measurements were conducted under standardized laboratory conditions, including controlled ambient temperature, low-light exposure, and minimized environmental noise. Participants were free either to remain awake or to fall asleep naturally during the intervention period. The control condition was included to assess the physiological effects of resting under a non-weighted blanket, whereas the weighted blanket conditions were designed to evaluate load-specific physiological and neurophysiological responses.

**Protocol B** was conducted in participants’ own homes under their habitual sleep environment. Prior to the initiation of the study, participants completed a series of questionnaires and measurements related to their overall health status. For the assessment of sleep architecture, participants were connected to a portable EEG/EOG system (HST-mit-tablet, SOMNOmedics AG, Randersacker, Germany). In addition, two iButton sensors were placed on each participant, one on the pectoralis major muscle and one on the quadriceps muscle, to continuously monitor skin temperature throughout the night. A third iButton sensor was positioned on the bedside table to record ambient room temperature during sleep. Vital signs, including heart rate and blood pressure, were assessed using an automated blood pressure monitor (BP5100 Bronze Upper Arm Blood Pressure Monitor, OMRON Healthcare Co., Ltd., Kyoto, Japan).

**Figure 1:**
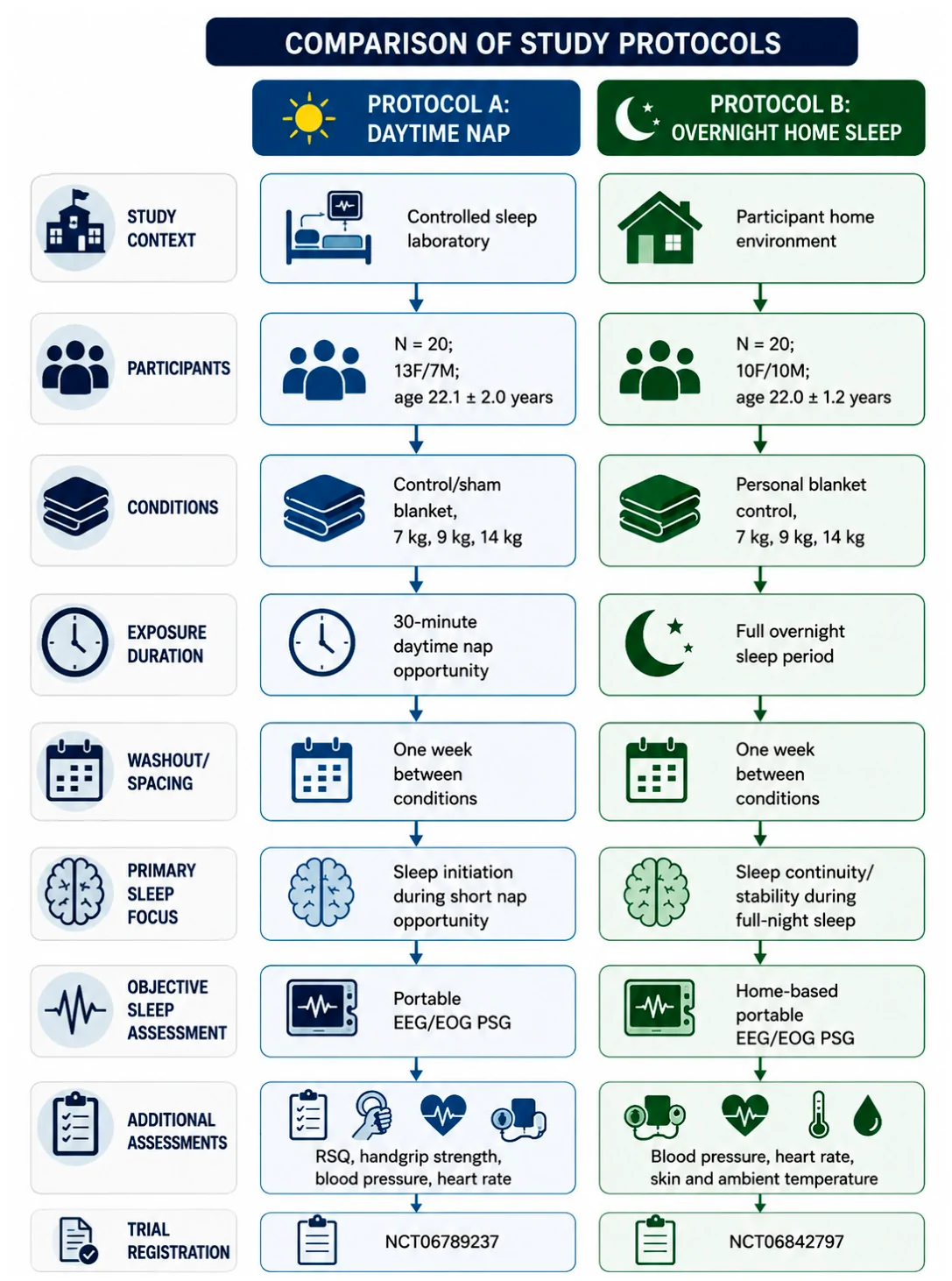
Study flow chart.

Each participant completed four overnight conditions, separated by one-week intervals: personal blanket (control), 7 kg weighted blanket, 9 kg weighted blanket, and 14 kg weighted blanket. The personal blanket was selected as the control condition because it represented each participant’s habitual and familiar bedding, thereby allowing the weighted blanket conditions to be compared against their usual home sleeping conditions. However, personal blankets were not standardized across participants in terms of weight, material, or thickness. The same three available blanket weights (7, 9, and 14 kg) were used as in Protocol A to allow the investigation of potential weight-dependent differences during overnight sleep. For each participant, another member of the research team used a random draw to assign the four blanket conditions to numbers 1–4. At the first visit, the participant stated their preferred order of the four numbers, and the blanket conditions were then administered according to the corresponding number-to-condition assignment. A new assignment was drawn for each participant. The one-week interval was used to separate successive acute overnight exposures to different blanket loads and to reduce the potential for carryover effects between experimental conditions. However, the adequacy of this interval for eliminating residual effects was not formally evaluated. Blood pressure measurements were obtained while participants were lying in bed immediately before sleep onset and again immediately upon awakening, prior to standing or engaging in any physical activity. Participants slept alone during all experimental nights and were free to sleep in their preferred sleeping position, with no restrictions imposed on posture throughout the intervention (Figure 1).

### 2.3. Measuring Instruments

#### 2.3.1. Body Composition

Body composition was assessed using anthropometric measurements, including BMI and bioimpedance (DC-360 S, TANITA Corporation, Tokyo, Japan), following standard methodology [15].

#### 2.3.2. Questionnaires

The following standardized questionnaires were administered using the interview method by experienced researchers. The Pittsburgh Sleep Quality Index (PSQI), a widely validated instrument for assessing subjective sleep quality and sleep disturbances, was used to evaluate sleep quality [16,17]. The 36-Item Short Form Health Survey (SF-36), a validated measure of health-related quality of life that has demonstrated satisfactory psychometric properties in Greek populations, was used to assess perceived physical and mental health [18,19]. The Beck Depression Inventory (BDI), which has demonstrated validity and reliability in Greek-language populations, was used to assess depressive symptoms [19,20]. Perceived stress was assessed using the Perceived Stress Scale (PSS), for which the Greek version has demonstrated satisfactory reliability and validity in the general Greek adult population [21,22].

#### 2.3.3. Sleep Architecture

A portable sleep monitoring system was used to assess sleep quality and quantity (Home Sleep Test, SOMNOmedics, GmbH, Germany). The HomeSleepTest REM+ is an ambulatory sleep-monitoring system designed for objective assessment of sleep staging and sleep continuity using EEG, EOG, and EMG recordings. Its portable configuration allows repeated sleep assessment with minimal participant burden and is suitable for recordings in both laboratory and habitual home environments [23]. The system records EEG, EOG, and EMG signals overnight. EEG data were analyzed in 30-second epochs using SOMNOmedics PSG analysis software (Domino panel ver. 3.0.0.8) with manual editing. The HomeSleepTest REM+ device was used for PSG, with electrodes placed according to the 10/20 system on key brain regions: Fp1 (left) and M1 for EEG, and near the eyes for EOG. This portable montage permitted assessment of sleep stages and continuity using the recorded EEG, EOG, and EMG signals, but provided fewer EEG recording sites than a full polysomnographic montage.

Analysis of the sleep study was reported as followed: Total Sleep Time (total amount of sleep time scored during the total recording time); Sleep Efficiency (Total sleep time/Time in bed); Sustained Sleep Efficiency (Total sleep time/(Time in bed–Sleep laten-cy stage 2); Sleep Latency (the period of time required to fall asleep after going to bed and attempting to initiate sleep); Sleep Latency N1 (the period of time between wakefulness and when sleep begins); Sleep Latency N2 (the period of time between time in bed and sleep onset stage 2); REM Latency (the amount of time elapsed between the onset of sleep to the first REM stage); Wakes (the number of awakenings during the sleep period or transitions to full wakefulness after sleep onset).

#### 2.3.4. Temperature

Skin temperature was measured at 1 min intervals throughout the overnight recording (at least 8 h) using wireless iButton temperature sensors (DS1921H, Maxim Integrated, San Jose, CA, USA). iButtons have previously demonstrated good accuracy and precision for human skin-temperature assessment and are considered suitable for laboratory and field measurements [24]. Sensors were positioned over the pectoralis major and quadriceps regions to provide repeated measurements from two proximal skin sites; these locations were not intended to represent a standardized whole-body mean skin-temperature protocol. A third iButton was positioned in the bedroom to record ambient temperature. For the present study, a two-site composite skin temperature was calculated as the arithmetic mean of the two recorded skin temperatures: Tskin = (Tchest + Tthigh)/2.

#### 2.3.5. Statistical Analysis

Statistical analyses were performed using IBM SPSS Statistics for Windows, version 29.0 (IBM Corp., Armonk, NY, USA). Baseline characteristics were summarized descriptively for each protocol. No inferential comparisons were performed between the two protocol cohorts because they comprised independent samples from methodologically distinct protocols. Baseline questionnaire scores were summarized using medians and interquartile ranges (25th–75th percentiles). Questionnaire scores were summarized using available observations, and sample sizes could differ across questionnaires because of missing data.

Repeated-measures General Linear Models were used to assess condition effects across the control, 7 kg, 9 kg, and 14 kg conditions within each protocol. For the overnight sustained sleep efficiency analysis, gender was included as a between-subjects factor. Each sleep outcome was analyzed separately. The assumption of sphericity was assessed using Mauchly’s test; when this assumption was violated, Greenhouse–Geisser-adjusted degrees of freedom and *p*-values were reported. Bonferroni-adjusted pairwise comparisons were used to examine condition differences where applicable. Effect sizes were estimated using partial eta squared (η^2^p). Within each blanket condition, pre-to-post changes in physiological and subjective outcomes were assessed using paired-samples *t*-tests, including participants with both measurements available for the respective outcome.

To assess the robustness of the reported findings, non-parametric sensitivity analyses were performed using the Friedman test for each sleep outcome across the four experimental conditions, including participants with complete data for the respective outcome. The repeated-measures analysis of overnight sustained sleep efficiency included only participants with a recorded value in all four blanket conditions. Of the 20 participants in Protocol B, seven met this complete-case criterion; the remaining 13 lacked at least one required observation.

For overnight sustained sleep efficiency, an additional sensitivity analysis was conducted using a repeated-measures GLM without gender as a between-subjects factor, retaining the same seven complete cases. The distributions of the within-participant differences between each weighted blanket condition and control were examined using the Shapiro–Wilk test for overnight sustained sleep efficiency. Given the small sample size, the normality test results were interpreted cautiously. Participants were also classified as good or poor sleepers according to PSQI cutoff criteria for exploratory subgroup analyses. The significance level was set at *p* ≤ 0.05. Because the two protocols were conceptually related but methodologically distinct, statistical inference was kept protocol-specific.

#### 2.3.6. Power Analysis

A retrospective sensitivity analysis was performed using G*Power version 3.1.9.2 [25] to assess the detectable effect sizes for the sample of 20 participants included in each of the two independent protocols. The calculation was based on a repeated-measures ANOVA (within-subject factors) with four experimental conditions, assuming a significance level of α = 0.05, a target statistical power of 85%, and a correlation of 0.50 among repeated measurements. Under the assumption of sphericity (ε = 1.00), the minimum detectable effect size was f = 0.287. When a more conservative nosphericity correction was applied (ε = 0.50), the minimum detectable effect size increased to f = 0.371. These estimates apply to the omnibus within-subject effect under the specified assumptions and do not establish the statistical power of individual pairwise comparisons. As the original sample size calculation could not be verified, the present sensitivity analysis should not be interpreted as an a priori justification of the sample size. Moreover, the overnight sustained sleep efficiency analysis included only seven participants with complete data across all four conditions. Therefore, the sensitivity estimates calculated for *n* = 20 do not apply to this outcome, for which the small analysis sample substantially limits the precision and robustness of the findings.

## 3. Results

Baseline characteristics are summarized descriptively for each protocol in Table 1. Both protocols enrolled healthy young adults with mean BMI in the normal range. In the daytime nap protocol, participants had moderate perceived stress and poor sleep quality on average. In the overnight protocol, females showed higher SF-36 total scores than males, while other questionnaire measures did not differ significantly by sex.

**Figure 2:**
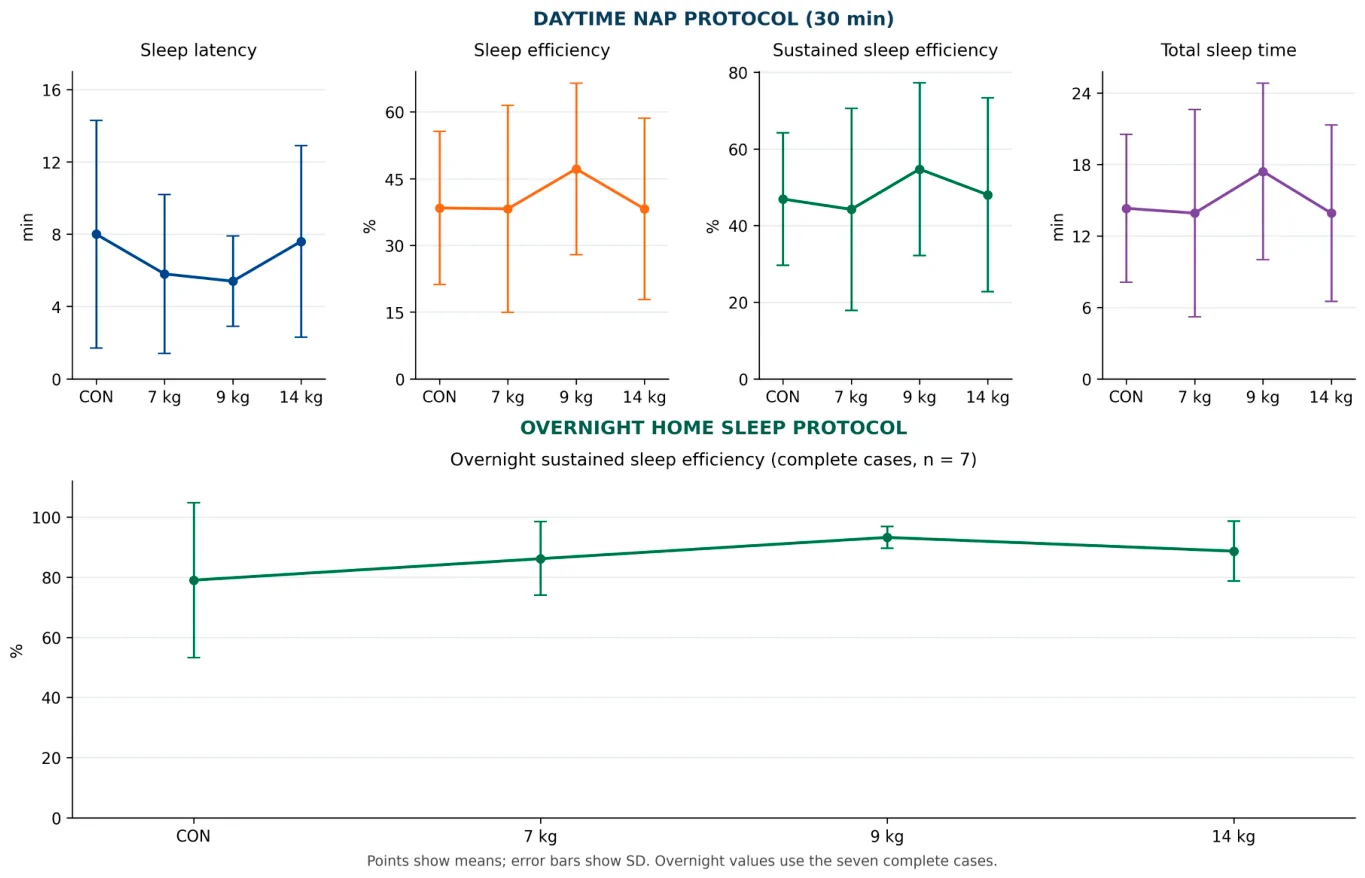
Main sleep outcomes by weighted blanket load. **Note:** Points represent means and error bars represent standard deviations. The overnight sustained sleep efficiency panel presents the seven participants with complete data across all four conditions. During the overnight home sleep protocol, the original repeated-measures GLM including gender as a between-subjects factor indicated a significant main effect of condition on sustained sleep efficiency [Greenhouse–Geisser-adjusted: F(1.929, 9.646) = 14.223, *p* = 0.001, η^2^p = 0.740]. Bonferroni-adjusted pairwise comparisons from this model indicated higher sustained sleep efficiency under the 9 kg (*p* = 0.004) and 14 kg (*p* = 0.002) conditions compared with control. However, a sensitivity analysis excluding gender, using the same seven complete cases, did not confirm a significant overall condition effect [F(3, 18) = 0.630, *p* = 0.605]. Therefore, given the small number of complete cases and the lack of confirmation in both sensitivity analyses, the significant findings from the original model should be regarded as exploratory and model-dependent rather than as robust evidence of differences between blanket conditions. A non-parametric Friedman sensitivity analysis also did not confirm a significant condition effect on overnight sustained sleep efficiency [χ^2^(3) = 3.000, *p* = 0.392].

No statistically significant differences were observed between conditions for daytime nap sleep variables (Table 2, Figure 2).

Although 20 participants were included in Protocol B, complete sustained sleep efficiency data across all four conditions were available for seven participants; the remaining 13 were excluded from the repeated-measures analysis for this outcome. In the overnight home protocol, the original repeated-measures GLM, which included gender as a between-subjects factor, indicated a significant main effect of condition on sustained sleep efficiency [Greenhouse-Geisser-adjusted: F(1.929, 9.646) = 14.223, *p* = 0.001, η^2^p = 0.740]. Bonferroni-adjusted pairwise comparisons from this model indicated significantly higher sustained sleep efficiency under both the 9 kg (*p* = 0.004) and 14 kg (*p* = 0.002) weighted blanket conditions compared with control. No significant difference between the 9 kg and 14 kg conditions was observed. However, a sensitivity analysis excluding gender as a between-subjects factor, while retaining the same seven complete cases, did not confirm a significant overall condition effect [F(3, 18) = 0.630, *p* = 0.605]. Shapiro–Wilk tests indicated departures from normality in the within-participant differences between control and the 7 kg (*p* = 0.045), 9 kg (*p* = 0.006), and 14 kg (*p* = 0.002) conditions (*n* = 7). A non-parametric Friedman sensitivity analysis also did not identify a statistically significant difference in overnight sustained sleep efficiency across the four conditions [χ^2^(3) = 3.000, *p* = 0.392]. Friedman sensitivity analyses of the remaining sleep outcomes presented in Table 2 likewise did not identify statistically significant differences across conditions (all *p* > 0.05). Given that the sustained sleep efficiency analysis was based on only seven complete cases and that the significant condition effect was not reproduced in either sensitivity analysis, this finding should be regarded as exploratory and model-dependent rather than as robust evidence of a condition effect. Sleep efficiency and total sleep time differed numerically across conditions but did not reach statistical significance (Table 2, Figure 2).

**Table 2:** Main sleep outcomes across blanket conditions in the two protocol components.

Outcome	CON	7 kg	9 kg	14 kg	*p*-Value
**Daytime Nap Protocol**					
TST (min)	14.3 ± 6.2	13.9 ± 8.7	17.4 ± 7.4	13.9 ± 7.4	0.122
Sleep efficiency (%)	38.4 ± 17.2	38.2 ± 23.3	47.2 ± 19.3	38.2 ± 20.4	0.130
Sustained sleep efficiency (%)	46.9 ± 17.3	44.2 ± 26.4	54.7 ± 22.5	48.0 ± 25.3	0.242
Sleep latency (min)	8.0 ± 6.3	5.8 ± 4.4	5.4 ± 2.5	7.6 ± 5.3	0.169
Sleep latency N2 (min)	13.9 ± 10.2	15.7 ± 9.3	11.0 ± 7.5	15.1 ± 10.3	0.368
Wakes (index)	5.1 ± 1.6	4.2 ± 1.5	4.1 ± 1.8	4.4 ± 1.6	0.147
**Overnight Home Sleep Protocol**					
TST (h)	6.1 ± 2.0	7.4 ± 1.4	7.5 ± 1.3	7.3 ± 0.5	0.188
Sleep efficiency (%)	79.4 ± 14.6	86.4 ± 11.0	89.2 ± 6.2	88.0 ± 6.9	0.168
Sustained sleep efficiency (%)	79.1 ± 25.7	86.2 ± 12.4	93.2 ± 3.6 *	88.7 ± 9.9 *	**0.001**
Sleep latency (min)	34.3 ± 28.5	10.9 ± 7.2	19.6 ± 25.0	16.8 ± 14.7	0.272
Sleep latency N2 (min)	36.3 ± 28.1	14.9 ± 7.4	20.5 ± 25.8	29.6 ± 42.3	0.554
Wakes (index)	25.7 ± 23.0	25.6 ± 28.0	20.6 ± 11.6	20.5 ± 12.7	0.671

**Notes:** Values are mean ± SD. * Significantly different from control condition following Bonferroni post hoc analysis (*p* ≤ 0.05): CON vs. 9 kg, *p* = 0.004; CON vs. 14 kg, *p* = 0.002. Significance level set at *p* ≤ 0.05. TST: total sleep time. The repeated-measures analysis of overnight sustained sleep efficiency was based on seven participants with complete observations across all four experimental conditions (*n* = 7). The reported *p*-value and Bonferroni-adjusted pairwise comparisons for overnight sustained sleep efficiency were obtained from the original repeated-measures GLM including gender as a between-subjects factor. Non-parametric Friedman sensitivity analyses were additionally performed for all 12 sleep outcomes using participants with complete data for each outcome; none identified a statistically significant condition effect (all *p* > 0.05). For overnight sustained sleep efficiency, neither the repeated-measures sensitivity analysis excluding gender (*p* = 0.605) nor the Friedman test (*p* = 0.392) confirmed the significant condition effect observed in the original model (*p* = 0.001). Therefore, the original significant finding and associated pairwise comparisons should be regarded as exploratory and model-dependent rather than as robust evidence of differences between blanket conditions.

In the daytime nap protocol, relaxation increased after lying down in all conditions, with the largest increase under the 9 kg blanket. Handgrip strength decreased after all weighted blanket conditions, consistent with a reduction in muscle tone or arousal. Heart rate and systolic blood pressure decreased significantly after the control and 7 kg conditions, with the weighted 7 kg condition showing a heart rate decrease of approximately 5.7% and a systolic pressure decrease of approximately 4.9%.

In the overnight protocol, systolic blood pressure decreased significantly after the 9 kg condition by approximately 4.3%. Heart rate and diastolic blood pressure did not significantly differ across conditions (Table 3).

Moreover, temperature outcomes remained stable across blanket loads and therefore do not appear to explain the numerical differences observed in overnight sleep-continuity measures (Table 4).

## 4. Discussion

The present integrated study examined the acute effects of different weighted blanket loads across two distinct sleep contexts. During the 30-min daytime nap protocol, no statistically significant differences in objective sleep outcomes were observed across blanket conditions. In the overnight home protocol, the original gender-adjusted model indicated a significant condition effect on sustained sleep efficiency, with higher values under the 9 kg and 14 kg conditions compared with control. However, this analysis was based on only seven participants with complete data, and the significant condition effect was not reproduced in either the sensitivity analysis excluding gender or the non-parametric Friedman analysis. Accordingly, this finding should be regarded as exploratory and model-dependent rather than as robust evidence that weighted blanket load improves overnight sustained sleep efficiency. In addition, systolic blood pressure decreased significantly from pre- to post-sleep following the 9 kg condition.

Overall, the present findings do not provide robust evidence that weighted blankets improve objective sleep outcomes across either sleep context. The isolated finding for overnight sustained sleep efficiency was based on a small complete-case sample and was sensitive to model specification, precluding firm conclusions regarding an effect of weighted blanket load on overnight sleep continuity. Daytime naps depend heavily on the ability to transition from wakefulness to sleep within a relatively short time window, whereas overnight sleep also depends on maintaining sleep continuity across a prolonged sleep period. In the present study, weighted blanket load did not significantly affect sleep initiation or other objective sleep outcomes during the 30-min nap opportunity. Although the original gender-adjusted analysis suggested a potential effect of weighted blanket load on overnight sustained sleep efficiency, the absence of a significant overall condition effect in the sensitivity analysis prevents a firm conclusion regarding improvements in sleep continuity. Larger studies are needed to determine whether weighted blanket effects differ between daytime napping and overnight sleep. Deep pressure stimulation is a plausible mechanism. Evenly distributed pressure may reduce physiological arousal, promote parasympathetic tone, and enhance the subjective sense of calm and security [9,10,26,27,28].

However, the present study was not designed to directly test these mechanisms. Although the original gender-adjusted model indicated a condition effect on overnight sustained sleep efficiency, this finding was not reproduced in the sensitivity analysis. Consequently, the present data do not establish a robust effect on sleep continuity or support conclusions regarding the underlying mechanisms. No statistically significant differences in temperature outcomes were observed across blanket conditions. Previous evidence of increased pre-sleep melatonin following weighted blanket use provides an additional potential pathway that warrants further investigation [12,29].

The present findings do not establish a definitive optimal blanket load. During the daytime protocol, no significant differences in objective sleep outcomes were detected across blanket conditions. During the overnight protocol, the original gender-adjusted model indicated higher sustained sleep efficiency under the 9 kg and 14 kg conditions compared with control. However, the overall condition effect was not reproduced in the sensitivity analysis excluding gender, and these findings should therefore be considered exploratory rather than conclusive evidence of a dose-dependent effect.

The 9 kg condition was also associated with a significant pre-to-post reduction in systolic blood pressure. Because blanket loads were fixed rather than individualized according to body mass, the present findings should not be interpreted as establishing a universal dose recommendation. Future studies should directly compare individualized weight-to-body-mass ratios in larger samples. The findings may be relevant in settings where non-pharmacological and low-burden sleep strategies are desirable, such as workplace rest opportunities, athletic recovery, shift-work recovery, stress-related sleep complaints, and home sleep hygiene. Nevertheless, the current participants were young and healthy, and the acute responses observed here should not be generalized directly to patients with chronic insomnia, anxiety disorders, obstructive sleep apnea, chronic pain, or older adults without further trials.

### Strengths and Limitations

Strengths of the integrated work include the use of objective polysomnography in both protocols, repeated exposure to multiple blanket loads, assessment of both subjective and physiological outcomes, and the inclusion of ecologically valid home-based overnight measurements. The study also addresses two distinct and clinically relevant sleep contexts: short daytime napping and habitual overnight sleep.

Several limitations should be acknowledged. First, the two protocol components were conducted in separate cohorts with different sex distributions, so the data should be interpreted as complementary rather than as one pooled sample. Second, sample sizes were small and limited to healthy young adults, which restricts the generalizability of the findings to older adults, individuals with different body compositions, and clinical populations. In particular, the present findings should not be extrapolated to individuals with insomnia, anxiety, chronic pain, or other relevant clinical conditions, as these populations were not represented in the study. Third, blanket loads were fixed rather than individualized according to body mass. Consequently, participants with different body masses may have received different relative levels of deep pressure stimulation, limiting the ability to identify an individualized or optimal dose.

However, commercially available fixed-weight blankets were intentionally used to reflect their pragmatic real-world application. Fourth, each protocol assessed only the acute response to a single sleep opportunity under each blanket condition. Therefore, the present study cannot determine the long-term efficacy or safety of repeated weighted blanket use, nor whether responses are maintained, attenuated, or altered over time due to habituation. Furthermore, the original sample size calculation could not be verified, and the retrospective sensitivity analysis does not replace an a priori sample size justification. The relatively small sample size may have limited the ability to detect smaller effects and differences in individual pairwise comparisons. Consequently, nonsignificant findings should not be interpreted as evidence of equivalence between experimental conditions. Longer-term studies with repeated exposure are needed to evaluate the durability of any effects, potential adaptation to weighted blanket use, and safety over extended periods. Finally, the portable PSG system used a limited EEG montage, with a frontal EEG recording site (Fp1) referenced to M1, alongside EOG and EMG recordings. Compared with a full polysomnographic montage, the reduced EEG coverage may limit the precision of sleep-stage classification and does not permit detailed assessment of regional brain activity. Thus, small differences in sleep architecture should be interpreted cautiously. In addition, the home-based protocol was conducted under participants’ usual domestic conditions. Ambient light and noise were not standardized across experimental nights, while ambient temperature was measured but not experimentally controlled. Variation in these conditions may have contributed to differences in sleep outcomes between sessions and may limit comparability between the home-based protocol and the controlled laboratory nap protocol.

Additionally, habitual light exposure and physical activity were not systematically monitored or standardized before the experimental sessions, and participants’ chronotype or circadian preference was not formally assessed. Although ambient lighting was controlled during the laboratory nap sessions, participants’ prior light exposure remained unmeasured. Light exposure, physical activity, and circadian preference may independently influence circadian timing, sleep pressure, and sleep initiation, potentially contributing to variability between participants and sessions, particularly during the daytime nap protocol. The use of participants’ habitual blankets as the control condition reflected their usual home sleeping conditions but introduced a separate lack of standardization, as blanket characteristics may have differed between participants. This variability should be considered when interpreting comparisons between the control and weighted blanket conditions. Furthermore, the overnight sustained sleep efficiency analysis was based on only seven complete cases, with markedly unequal gender subgroup sizes (one versus six participants). This imbalance, together with the small analysis sample and substantial interindividual variability, limits the precision and robustness of the statistical estimates. Importantly, the significant condition effect observed in the original gender-adjusted model was not reproduced in either the sensitivity analysis excluding gender or the non-parametric Friedman analysis. Thus, the statistical inference for sustained sleep efficiency was sensitive to model specification and was not robust across analytical approaches. Consequently, this finding should be regarded as exploratory and model-dependent rather than as confirmatory evidence that weighted blanket load improves overnight sleep continuity. Future studies should objectively monitor or standardize pre-sleep light exposure and physical activity and assess chronotype to better account for circadian variability when evaluating weighted blanket effects.

## 5. Conclusions

These two randomized crossover studies examined the acute effects of different blanket loads on objective sleep outcomes during daytime napping and overnight home sleep in healthy young adults. No statistically significant differences in objective sleep outcomes were observed across blanket conditions during the 30-min daytime nap protocol. During overnight home sleep, the original gender-adjusted model indicated a significant condition effect on sustained sleep efficiency, with higher values under the 9 kg and 14 kg conditions compared with control. However, this analysis was based on only seven participants with complete data; sustained sleep efficiency was the only significant objective sleep outcome in the overnight study, and the condition effect was not confirmed by either the sensitivity analysis excluding gender or the non-parametric Friedman analysis. Accordingly, the sustained sleep efficiency finding should be regarded as exploratory and model-dependent rather than as confirmatory evidence that weighted blanket load improves overnight sleep. The 9 kg condition was also associated with a pre-to-post reduction in systolic blood pressure. Given the small sample sizes, the absence of significant effects across the other objective sleep outcomes, the discrepancy between the original and sensitivity analyses, and the acute nature of the protocols, these findings should be considered preliminary and do not establish an overall beneficial effect of weighted blankets on sleep. Larger, adequately powered, and longer-term studies are needed to determine whether weighted blankets provide reproducible benefits for specific sleep outcomes and to establish whether such effects extend to clinical populations.

## Figures and Tables

**Table 1:** Baseline characteristics of participants in the two protocol components.

Variable	Protocol A: Daytime Nap	Protocol B: Overnight Home Sleep
**N**	20	20
**Sex**	7M/13F	10M/10F
**Age (years)**	22.0 ± 1.9	22.0 ± 1.2
**Height (cm)**	172.9 ± 10.9	171.4 ± 9.6
**Body weight (kg)**	69.7 ± 12.2	68.5 ± 12.7
**BMI (kg/m^2^)**	23.1 ± 1.7	23.3 ± 3.6
**Body fat (%)**	18.3 ± 7.3	18.8 ± 8.6
**Muscle mass (kg)**	54.1 ± 12.2	52.8 ± 9.8
**PSQI**	6.00 (4.00–8.00)	5.00 (3.00–7.00)
**AIS-8**	4.50 (3.00–6.75)	6.50 (2.50–11.00)
**SF-36 total score (%)**	82.50 (76.75–86.25)	91.00 (85.50–94.00)
**BDI**	4.50 (2.50–8.00)	5.50 (2.25–9.50)
**PSS**	19.50 (17.75–22.25)	19.00 (18.00–21.75)

**Notes:** Values are mean ± SD unless otherwise indicated. BDI: Beck Depression Inventory; PSQI: Pittsburgh Sleep Quality Index; PSS: Perceived Stress Scale; AIS-8: Athens Insomnia Scale. Values are presented as mean ± SD for demographic and anthropometric characteristics and as median (25th–75th percentile) for questionnaire scores. Both protocols included 20 participants. Questionnaire scores were summarized using available observations; the number of observations may differ between questionnaires because of missing data.

**Table 3:** Summary of key physiological and subjective responses.

Protocol	Outcome	CON	7 kg	9 kg	14 kg	Interpretation
**Daytime nap**	Δ% RSQ	+17.8 ± 17.6	+17.0 ± 13.7	+20.3 ± 17.1	+13.7 ± 21.3	Positive mean changes in relaxation were observed across conditions
**Daytime nap**	Δ% Handgrip	+6.9 ± 10.8	−9.2 ± 12.8	−10.2 ± 8.6	−10.3 ± 13.5	Mean handgrip values decreased following the weighted blanket conditions
**Daytime nap**	Δ% Heart rate	−6.1 ± 7.5	−5.7 ± 8.1	-	-	Significant pre-post reductions were observed for CON and 7 kg
**Daytime nap**	Δ% Systolic pressure	−5.6 ± 12.7	−4.9 ± 10.5	-	-	Significant pre-post reductions were observed for CON and 7 kg
**Overnight home sleep**	Δ% Systolic pressure	0.0 ± 0.9	−1.7 ± 1.0	−4.3 ± 1.2	−0.8 ± 0.7	Significant pre-post reduction was observed following the 9 kg condition
**Overnight home sleep**	Δ% Heart rate	−0.1 ± 9.9	−2.6 ± 11.2	+2.9 ± 8.6	+0.9 ± 7.5	No significant condition effect was observed

**Notes:** RSQ: Relaxation State Questionnaire; Δ%: percent change from pre- to post-condition–indicates that the corresponding descriptive value was not available for inclusion in this summary table.

**Table 4:** Overnight skin and ambient temperature outcomes.

Variable	CON	7 kg	9 kg	14 kg	*p*-Value
**Ambient temperature (°C)**	20.0 ± 1.2	19.3 ± 1.2	19.7 ± 0.6	20.2 ± 0.6	0.233
**Chest temperature (°C)**	26.6 ± 1.5	26.2 ± 1.8	25.4 ± 1.7	25.2 ± 2.5	0.383
**Leg temperature (°C)**	26.8 ± 1.5	26.0 ± 0.7	26.1 ± 2.2	25.6 ± 1.1	0.403

**Notes:** Values are mean ± SD. Significance level: *p* ≤ 0.05. No significant differences were observed across blanket conditions.

## Data Availability

The original contributions presented in the study are included in the article; further inquiries can be directed to the corresponding author.

## References

[1] Chattu V.K., Manzar M.D., Kumary S., Burman D., Spence D.W., Pandi-Perumal S.R. (2019). The global problem of insufficient sleep and its serious public health implications. Healthcare.

[2] Itani O., Jike M., Watanabe N., Kaneita Y. (2017). Short sleep duration and health outcomes: A systematic review, meta-analysis, and meta-regression. Sleep Med..

[3] Hafner M., Stepanek M., Taylor J., Troxel W.M., Van Stolk C. (2017). Why sleep matters—The economic costs of insufficient sleep: A cross-country comparative analysis. Rand Health Q..

[4] Åkerstedt T., Garefelt J., Richter A., Westerlund H., Hanson L.L.M., Sverke M., Kecklund G. (2015). Work and sleep-A prospective study of psychosocial work factors, physical work factors, and work scheduling. Sleep.

[5] Chaput J.P., Dutil C., Featherstone R., Ross R., Giangregorio L., Saunders T.J., Janssen I., Poitras V.J., Kho M.E., Ross-White A. (2020). Sleep timing, sleep consistency, and health in adults: A systematic review. Appl. Physiol. Nutr. Metab..

[6] Dutheil F., Danini B., Bagheri R., Fantini M.L., Pereira B., Moustafa F., Trousselard M., Navel V. (2021). Effects of a short daytime nap on the cognitive performance: A systematic review and meta-analysis. Int. J. Environ. Res. Public Health.

[7] Simpson N.S., Gibbs E.L., Matheson G.O. (2017). Optimizing sleep to maximize performance: Implications and recommendations for elite athletes. Scand. J. Med. Sci. Sports.

[8] Desai D., Momin A., Hirpara P., Jha H., Thaker R., Patel J. (2024). Exploring the Role of Circadian Rhythms in Sleep and Recovery: A Review Article. Cureus.

[9] Mullen B., Champagne T., Krishnamurty S., Dickson D., Gao R.X. (2008). Exploring the safety and therapeutic effects of deep pressure stimulation using a weighted blanket. Occup. Ther. Ment. Health.

[10] Champagne T., Mullen B., Dickson D., Krishnamurty S. (2015). Evaluating the safety and effectiveness of the weighted blanket with adults during an inpatient mental health hospitalization. Occup. Ther. Ment. Health.

[11] Ekholm B., Spulber S., Adler M. (2020). A randomized controlled study of weighted chain blankets for insomnia in psychiatric disorders. J. Clin. Sleep Med..

[12] Meth E.M.S., Brandão L.E.M., van Egmond L.T., Xue P., Grip A., Wu J., Adan A., Andersson F., Pacheco A.P., Uvnäs-Moberg K. (2023). A weighted blanket increases pre-sleep salivary concentrations of melatonin in young, healthy adults. J. Sleep. Res..

[13] Yu J., Du J., Yang Z., Chen W., Sun S., Gan M., Cai Y., Zhang L., Sun K., Xu J. (2024). Effect of weighted blankets on sleep quality among adults with insomnia: A pilot randomized controlled trial. BMC Psychiatry.

[14] Zisapel N. (2018). New perspectives on the role of melatonin in human sleep, circadian rhythms and their regulation. Br. J. Pharmacol..

[15] Masakul P., Rungsihirunrat K. (2020). The Effectiveness of 3-Month Exercise Program on Body Composition in Overweight Adult Army Male Officers at Support Unit of Armed Forces Development Command. Int. J. Multidiscip. Res. Publ. (IJMRAP).

[16] Buysse D.J., Reynolds C.F., Monk T.H., Berman S.R., Kupfer D.J. (1989). The Pittsburgh Sleep Quality Index: A new instrument for psychiatric practice and research. Psychiatry Res..

[17] Kotronoulas G.C., Papadopoulou C.N., Papapetrou A., Patiraki E. (2011). Psychometric evaluation and feasibility of the Greek Pittsburgh sleep quality index (GR-PSQI) in patients with cancer receiving chemotherapy. Support. Care Cancer.

[18] Ware J.E. (2000). SF-36 Health Survey update. Spine.

[19] Pappa E., Kontodimopoulos N., Niakas D. (2005). Validating and norming of the Greek SF-36 Health Survey. Qual. Life Res..

[20] Beck A.T., Guth D., Steer R.A., Ball R. (1997). Screening for major depression disorders in medical inpatients with the Beck Depression Inventory for Primary Care. Behav. Res. Ther..

[21] Wang Z., Chen J., Boyd J.E., Zhang H., Jia X., Qiu J., Xiao Z. (2011). Psychometric properties of the Chinese version of the perceived stress scale in policewomen. PLoS ONE.

[22] Andreou E., Alexopoulos E.C., Lionis C., Varvogli L., Gnardellis C., Chrousos G.P., Darviri C. (2011). Perceived Stress Scale: Reliability and validity study in Greece. Int. J. Environ. Res. Public Health.

[23] Davidson S., Carter J.F., Stanyer E.C., Sharman R., Roman C., Kyle S.D., Tarassenko L. (2026). Longitudinal cardiorespiratory wearable sleep staging in the home. Front. Neurosci..

[24] van Marken Lichtenbelt W.D., Daanen H.A.M., Wouters L., Fronczek R., Raymann R.J.E.M., Severens N.M.W., Van Someren E.J.W. (2006). Evaluation of wireless determination of skin temperature using iButtons. Physiol. Behav..

[25] Faul F., Erdfelder E., Lang A.G., Buchner A. (2007). G* Power 3: A flexible statistical power analysis program for the social, behavioral, and biomedical sciences. Behav. Res. Methods.

[26] Payne D.R., Vinson J., Powers J., McDaniel B.T., Sevier C., Marshall C., Sell S. (2024). Effect of Weighted Blanket Versus Traditional Practices on Anxiety and Pain in Patients Undergoing Elective Surgery: A Multicenter Randomized Controlled Trial. AORN J..

[27] Dickson D.A., Gantt L., Swanson M. (2023). Effectiveness of the Weighted Blanket with Psychiatric Patients in the Emergency Department: A Pilot Study. J. Am. Psychiatr. Nurses Assoc..

[28] Baumgartner J.N., Quintana D., Leija L., Schuster N.M., Bruno K.A., Castellanos J.P., Case L.K. (2022). Widespread Pressure Delivered by a Weighted Blanket Reduces Chronic Pain: A Randomized Controlled Trial. J. Pain.

[29] Cederlund M., Steingrimsson S., Odéus E., Nyström K., Helgesson C., Sondell J.K., Franzén S., Opheim A. (2023). The introduction of a weighted blanket was not associated with a reduced collection of pharmaceuticals prescribed for sleep disturbances in children: A register-based study. Sleep Med..

